# eHealth and mHealth in Antimicrobial Stewardship to Reduce Mortality in Empirical Antimicrobial Therapy and a Systematic Review with a Meta-Analysis of Adequate Therapy

**DOI:** 10.3390/idr16040054

**Published:** 2024-08-01

**Authors:** Felipe Francisco Tuon, Tiago Zequinao, Marcelo Silva da Silva, Kleber Oliveira Silva

**Affiliations:** Laboratory of Emerging Infectious Diseases, Pontifícia Universidade Católica do Paraná, Curitiba 80215-901, Brazil; zequinao.tiago@hospitalcajuru.com.br (T.Z.); m.silva10@grupomarista.org.br (M.S.d.S.); kosilva@beckman.com (K.O.S.)

**Keywords:** antimicrobial stewardship, rapid diagnostic methods, mHealth, eHealth, antimicrobial resistance, pharmacotherapeutic algorithms

## Abstract

The urgent requirement for swift diagnostic methods in pathogen identification and antimicrobial susceptibility testing is emphasized by rising bacterial resistance and limited treatment options, which are particularly critical in sepsis management. The shift from traditional phenotype-based methods to rapid molecular and mass spectrometry techniques has significantly reduced result turnaround times, enhancing patient outcomes. In this systematic review with meta-analysis, the aspects of correct empirical antimicrobial therapy are evaluated to determine their impact on mortality. We performed a systematic review and meta-analysis on EMBASE, the Cochrane Library, Web of Science, and MEDLINE. Studies evaluating mortality associated with empirical adequate and inadequate therapy in different sites of infection were included. Outcomes included clinical cures in microbiologically evaluable patients. Among the sites of infection, the most studied were bloodstream infections (*n* = 9), followed by respiratory tract infections (*n* = 5), intra-abdominal infections (*n* = 5), and urinary tract infections (evaluated by 3 studies). Inadequate therapy was associated with an increase in mortality between 11 and 68%. Technologies to speed up pathogen identification are extremely necessary to reduce mortality.

## 1. Introduction

The global recognition of the need for rapid diagnostic measures in pathogen identification and antimicrobial susceptibility testing is driven by the limited availability of therapeutic options [1,2]. The effectiveness of antimicrobials against bacteria in infections has quickly evolved, primarily because of the rise in bacterial resistance and the simultaneous production of resistance genes. This situation particularly limits treatment options for patients suffering from sepsis [3,4,5,6,7]. In the treatment of sepsis, it is crucial to begin with empirical, rapid, and broad-spectrum antibiotic therapy. Once culture results and antimicrobial susceptibility testing are obtained, the therapy should be reassessed to consider de-escalation. This means narrowing the antimicrobial spectrum to reduce the selection pressure for multidrug-resistant bacteria [8]. However, improper or delayed prescription significantly raises patient morbidity and mortality rates. A retrospective study conducted in the United States between 2011 and 2014 found that delays in administering appropriate therapy were linked to poorer clinical outcomes. These included a 70% increase in the length of hospital stays, a 65% rise in total hospital costs, and a 20% higher risk of mortality [9], demonstrating how rapid and accurate diagnosis is essential for proper treatment.

For many years, the identification of microorganisms in clinical samples relied on determining phenotypic growth characteristics through biochemical tests. However, since the 1970s, the introduction of commercial tests combined with automated instruments has been used in routine laboratory work. These advancements, which include additional biochemical tests and standardized test readings, have enhanced identification accuracy and shortened the time required to release results. With the development of molecular tests, particularly polymerase chain reaction (PCR), regarded as the gold standard for microorganism identification, and the recent availability of mass spectrometry tests, the time to obtain results has become even shorter, significantly impacting patient morbidity and mortality [10,11,12,13]. However, the swift interaction between the laboratory and the clinician must be effective. Many clinicians, despite receiving laboratory information, often lack the expertise to make the best antimicrobial decisions due to the complexity involved in choosing the right antimicrobials. This is where antimicrobial stewardship becomes crucial.

mHealth, or mobile health, refers to the practice of medicine and public health supported by mobile devices such as smartphones, tablet computers, personal digital assistants, and other wireless devices [14]. It includes various applications such as health information delivery, patient monitoring, telemedicine, health education, disease surveillance, and health management, utilizing technologies like mobile apps, SMS messaging, and wearable devices. mHealth seeks to enhance healthcare services, patient outcomes, and access to health information and services, particularly targeting underserved populations in remote or resource-limited areas. eHealth, or electronic health, broadly refers to the use of information and communication technologies in healthcare. This encompasses a wide range of processes and services, including electronic health records, telehealth, telemedicine, health information systems, digital health interventions, patient portals, and the use of the internet and mobile devices for health education and information. The aim of eHealth is to improve the quality, efficiency, and accessibility of healthcare services, facilitate better communication between healthcare providers and patients, enhance the management of medical records and data, and support public health initiatives through more effective and efficient use of health information and technologies [15].

mHealth and eHealth have significant potential within antimicrobial stewardship that needs to be better utilized. In this review, we will compile some examples of the application of mHealth and eHealth in the routine management of antimicrobials, including tele-stewardship. After a brief narrative review, the aim of this study was to perform a systematic review with a meta-analysis to evaluate the effect of antimicrobial therapy adequacy on mortality.

## 2. The Importance of Antimicrobial Stewardship

The appropriate prescribing of antimicrobials (ATMs) in hospitals is crucial for both individual therapeutic success and patient safety. On a broader scale, it is vital for controlling multidrug-resistant microorganisms and managing supply costs [16,17,18]. Selecting the most suitable ATM for treating bacterial and fungal infections, particularly healthcare-associated infections, is a complex process that involves multiple variables. When scaled to a hospital level, this process demands considerable time and a well-trained multidisciplinary team.

In both empirical and targeted treatment phases, there are significant challenges in managing infections. Empirical therapy relies on the clinician’s diagnostic expertise to identify the infection site and potential pathogens, utilizing local epidemiological data and understanding of microbial resistance to enhance predictive accuracy regarding the causative agents. Conversely, targeted treatment encounters difficulties related to the collection of samples and the need for rapid and reliable microbiological diagnosis, which are essential for promptly narrowing the antibiotic spectrum [19]. Additionally, in both phases of treatment, it is essential to consider various factors related to the patient, such as age, pregnancy and lactation status, allergies, and renal and hepatic function. Furthermore, drug-specific factors must be evaluated, including pharmacokinetics, tissue distribution, and the availability of medications within the local therapeutic arsenal [20,21].

Considering these challenges, antimicrobial stewardship programs have been implemented in hospitals in developed countries and have gained traction in other regions over the past decade. These programs aim to optimize the use of antimicrobials to improve patient outcomes and combat resistance [22]. These teams, typically consisting of an infectious disease physician, clinical pharmacist, and clinical microbiologist, implement structured initiatives to prevent and treat infections while auditing antimicrobial prescriptions. Their objective is to tailor antimicrobial therapy to individual patient needs. Numerous studies assessing the effectiveness of antimicrobial stewardship program interventions in both hospital and outpatient settings have been published, demonstrating predominantly positive outcomes in clinical, economic, and epidemiological domains [23,24,25,26,27].

## 3. Artificial Intelligence and Antimicrobial Stewardship

The initial application of artificial intelligence in healthcare emerged in the field of infectious diseases. Long before “antimicrobial stewardship” was formally defined in the scientific literature, an auxiliary system was developed to aid in diagnosing infections and optimizing antimicrobial therapy prescriptions. Known as MYCIN, this expert AI system was created in 1975 and utilized predefined rules established by programmers to mimic clinical reasoning. By analyzing patient records and microbiological laboratory data, MYCIN was able to assess the likelihood of specific microorganisms and suggest appropriate antimicrobial treatment options [28]. Despite demonstrating superior performance compared to physicians in tests and effectively reducing unnecessary antimicrobial use, MYCIN was ahead of its time. Technological limitations, such as the time required to input data into the system and the lack of personal computers, alongside ethical considerations, hindered its adoption in clinical practice [29].

With the advancement and widespread use of personal computers and mobile devices in recent years, machine learning has become a part of our daily lives, evident in streaming services and targeted advertisements based on user preferences. In the medical field, the easy capture and digitization of data have opened up numerous opportunities for leveraging this technology [30].

AI refers to the ability of a computerized system to mimic human decision-making processes. In healthcare, it exists on a spectrum: at one end are systems with fixed architectures, relying on clear rules and manually programmed formulas, such as evidence-based flowcharts or professional knowledge, exemplified by Expert Systems like MYCIN, which used approximately 600 predefined rules to simulate the decision-making of infectious disease specialists. Conversely, at the other end are machine learning systems, including deep learning, which autonomously develop algorithms based on patterns in data, often without programmer intervention but requiring large datasets. For instance, Google’s Diabetic Retinopathy project utilized a database of retinography images to create an algorithm capable of diagnosing diabetic retinopathy with sensitivity and specificity comparable to that of ophthalmologists [31,32]. Thus, artificial intelligence tools operate along a spectrum of varying dependence on human input, with the complexity of the generated algorithms being inversely related to this dependence. Deep learning algorithms, in particular, often become so intricate that they can be challenging to interpret, leading to what is referred to as a “black box” phenomenon in which the decision-making process is not easily understandable [30].

By 2019, in the field of infectious diseases, 60 CDSSs (Clinical Decision Support Systems) were reported in the scientific literature. Most of these tools were made focusing on bacterial diseases (63%), for use in ICUs (40%), and as infectious disease consultancy (25%), with the goal of diagnosing and predicting sepsis (66%), in high-income countries (90%). To date, only seven systems have been published in low- and middle-income countries, with six focused on HIV and tuberculosis. Evidence regarding the impact of CDSSs on clinical practice remains limited. Of these tools, only three have undergone clinical trials, while the others were evaluated solely for their performance metrics [31].

A randomized clinical trial evaluating a machine learning-based CDSS for sepsis diagnosis found that care and treatment were initiated significantly earlier in the machine learning-assisted group compared to the standard electronic system. Specifically, the administration of antimicrobial therapy and blood culture collection occurred, on average, 2.76 and 2.79 h earlier, respectively. Consequently, the trial reported a decrease in patient hospital stay from 13.0 to 10.7 days, a reduction of 6.3 h in average ICU stay, and a 12.4% decrease in hospital mortality within the machine learning group [33].

In a multicenter study utilizing predictive models for selecting antimicrobial therapy for the empirical treatment of bacteremia caused by Gram-negative bacteria, demographic and laboratory data were analyzed. Prediction models were developed based on the progression of microbiological examination results. The study reported an area-under-the-curve performance ranging from 0.63 to 0.85 when treatment was guided solely by the Gram stain and from 0.64 to 0.95 when guided by the identified pathogen, suggesting moderate-to-high performance. Furthermore, the findings indicated that even when compared to culture-guided ATM selection, the predictive model based on the pathogen led to the use of narrow-spectrum antibiotics [34,35].

In another randomized clinical trial comparing ATM choices for empirical treatment guided by an antimicrobial stewardship program with a clinical decision support system and clinical protocols versus those selected by the attending physician, several clinical outcomes were assessed. These included average hospital stay, readmission rates, and 30-day mortality, as well as outcomes related to multidrug-resistant organisms, such as *Clostridioides difficile* infection and multidrug-resistant Gram-negative bacteria, along with the cost implications of ATM. The results indicated no significant differences in outcomes between using the CDSS and not using it. However, when analyzing specific infection types, the intervention by the antimicrobial stewardship program led to a reduction in hospital stay of 0.53 days for cellulitis and a decreased mortality risk (OR 0.58) for community-acquired pneumonia [36].

## 4. Diagnostic Stewardship

**Figure 1:**
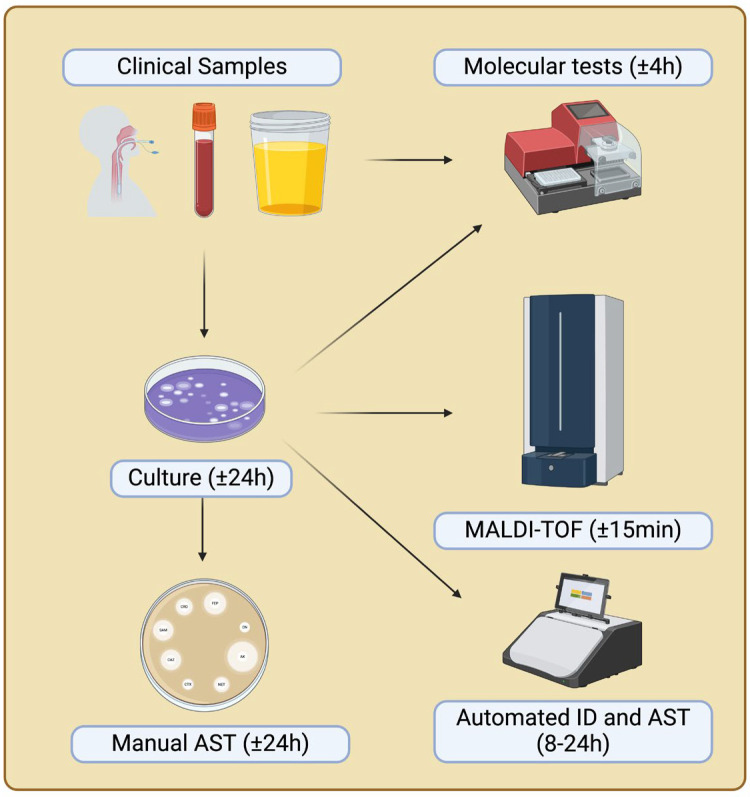
Flow of clinical sample processing in the microbiology laboratory and approximate time for results. The arrows indicate potential test pathways, including direct molecular examination of clinical samples or analysis following growth on culture media. MALDI-TOF (matrix-assisted laser desorption ionization time-of-flight); AST (antimicrobial susceptibility test); ID (identification).

Technological advancements have allowed clinical microbiology laboratories to adopt rapid microorganism identification methods, particularly through mass spectrometry techniques such as matrix-assisted laser desorption/ionization time-of-flight (MALDI-TOF) (see Figure 1). The effectiveness of this technology is due to its ability to identify a wide range of organisms, the rapid turnaround time for results (within minutes), the precision and reproducibility of the method, and its cost-effectiveness [37]. However, advances in molecular methods have introduced equipment capable of identifying different microorganisms present in a sample using the PCR multiplex technique [38,39,40,41,42]. Not only clinical laboratories but also public health reference laboratories can investigate hard-to-identify microorganisms using molecular technologies, such as sequencing the 16S rDNA gene, which is representative of prokaryotic organisms [43].

Like identification, antimicrobial susceptibility tests are essential for the clinical management of patients with infectious diseases, as well as in epidemiology, public health, new antimicrobial development, and clinical microbiology. However, many antimicrobial susceptibility tests currently in use were developed over 50 years ago and rely on phenotypic tests based on microbial growth, which typically require 48–72 h from the time of culture collection [2]. While various methodologies are being described and tested, the current gold standard for antimicrobial susceptibility test remains manual broth microdilution, as recommended by several standardization bodies. The primary methods for conducting antimicrobial susceptibility tests include disk diffusion, concentration gradient diffusion, and automated testing systems.

Advancements in microbiological methodologies directly affect the operational costs of performing tests. Nevertheless, all these tools are designed to optimize and enhance infection management, ultimately improving patient outcomes and supporting diagnostic and antimicrobial administration programs [44]. The use of these tools is closely linked to the concept of Diagnostic Stewardship, which involves modifying the medical process for requesting microbiological examinations, executing tests, and reporting results. This approach aims to enhance the treatment of infections and other medical conditions [45]. In the context of Diagnostic Stewardship, implementing measures to preserve antimicrobial use and optimize empirical therapy can be facilitated by utilizing the Cumulative Antibiogram. This tool is designed to guide initial empirical therapy decisions for treating infections in patients who do not yet have microbiological test results available to inform treatment [46]. The Cumulative Antibiogram is a report that accumulates combined data from microbiological examinations performed over a specified period, describing the percentage of microorganisms sensitive to selected antimicrobials [47], as well as the possibility of obtaining the minimum inhibitory concentration (MIC) to inhibit the growth of 50% and 90% of the organisms, MIC 50 and MIC 90, respectively [46].

Beyond empirical therapy, cumulative data are essential for monitoring resistance trends over time, conducting surveillance of antimicrobial-resistant organisms, and identifying areas for intervention through hospital infection prevention programs and antimicrobial stewardship. This approach is not restricted to a specific clinical isolate or hospital area, making it applicable to both individual institutions and broader regions [47,48,49,50]. The Clinical and Laboratory Standards Institute’s M39 document provides guidelines for calculating and compiling Cumulative Antibiogram data, including establishing confidence intervals, determining statistical significance, estimating percentiles, and calculating MIC 50 and MIC 90. Currently, LabPro software 5.0 (Beckman Coulter Inc., Sacramento, CA, USA) facilitates the automated generation of Cumulative Antibiograms, displaying percentages of growth inhibition across various concentrations of available antibiotics by panels, while also allowing for the assessment of MIC 90 and intermediate MICs [51].

## 5. The Choice of ATMs

The choice of antibiotics is a crucial aspect of treating bacterial infections and requires careful consideration to ensure therapeutic efficacy while minimizing the development of antimicrobial resistance. Several essential steps are necessary for the appropriate selection of antibiotics. The first step in selecting an antibiotic is making an accurate diagnosis of the infection. This involves identifying the infection site and, whenever possible, the specific pathogen responsible. Diagnostic methods may include clinical evaluations, bacterial cultures, imaging studies, and molecular tests. Once the pathogen is identified, it is essential to assess its sensitivity to antibiotics. Antimicrobial susceptibility tests, such as the disk diffusion method or broth microdilution, offer valuable insights into which antibiotics are effective against the specific pathogen. The selection of an antibiotic should consider its spectrum of activity, which refers to the range of organisms it can effectively target. For infections caused by identified specific pathogens, a narrow-spectrum antibiotic is preferred to minimize disruption to the body’s normal microbiota and reduce the risk of resistance development. In cases of severe infections or when the pathogen is unknown, a broad-spectrum antibiotic may be necessary initially.

It is essential to consider the pharmacokinetics of the antibiotic, including how it is distributed, metabolized, and eliminated by the body, as well as its mechanism of action and the relationship between drug concentration and bactericidal effect (pharmacodynamics). Factors such as the antibiotic’s ability to achieve therapeutic concentrations at the site of infection and potential drug interactions are also critical in the selection process. The selection of an antibiotic should also consider individual patient characteristics, such as allergies to antibiotics, comorbidities, renal and hepatic function, age, pregnancy, and lactation. Additionally, factors such as treatment cost and the ease of administration (e.g., oral versus intravenous) may also influence the choice of therapy.

Adherence to the principles of antimicrobial stewardship is essential throughout the antibiotic selection process to promote responsible use of these drugs, maximize therapeutic efficacy, protect public health, and minimize the development of antimicrobial resistance [52]. This is clearly translated in the meta-analysis we conducted evaluating the literature on the % of error in empirical antibiotic therapy.

## 6. Systematic Review with Meta-Analysis

### 6.1. Search Strategy

We searched EMBASE, the Cochrane Library, Web of Science, and MEDLINE for relevant English/Spanish/Portuguese/French/Deutsch-language articles published between 2012 and 2024. To find additional relevant research, we examined the sources cited in the reviews (systematic reviews with or without meta-analysis). The search criteria included terms from the following Medical Subject Headings: (antibiotic OR antimicrobial OR antibacterial) AND (adequate OR inadequate OR appropriate OR inappropriate OR incorrect OR correct). The complete query search is described in the Supplementary Materials (Table S1).

### 6.2. Data Collection Process

Two authors conducted a full-text analysis on the publications that passed the first abstract screening. We included studies with all of the following: (a) adult hospitalized patients; (b) diagnosis of any infections; (c) evaluation of adequacy of empirical therapy following microbiological culture of the site infection; and (d) evaluation of global mortality. Observational cohort studies, randomized controlled trials, and analyses of prospectively obtained data on the optimal timing of antibiotic administration were included. Meta-analyses, animal research, opinion pieces, small-scale studies, and editorial letters were not considered. Studies without site infection definitions were excluded too.

### 6.3. Data Items

We documented the participant selection procedure, inclusion criteria, study duration and time, study site (emergency room, intensive care unit, ward), study type, and sample size for each infection site. Data on the baseline and the time intervals of empirical antibiotic and adequacy (or not) according to culture were not evaluated due to extensive variability among each patient and study. When microbiological documentation of infection was available, these data were also considered.

### 6.4. Subgroup Evaluation

Subgroup analyses were conducted to determine whether antimicrobial adequacy was different among different site infections, including bloodstream infections, respiratory tract infections, abdominal infections, urinary tract infections, and sepsis. In the case of sepsis, the site of infection was not included in the analysis. 

### 6.5. Outcomes Assessment

All-cause mortality was the primary outcome measure. The mortality was variable, which included 30 days or during hospitalization (global). Infection-associated mortality was not considered once there are no criteria for this outcome.

### 6.6. Statistical Analysis

All statistical analyses were performed with Review Manager Version 5.3. Dichotomous data are presented as odds ratios (ORs) with 95% confidence intervals (CIs). Statistical heterogeneity among studies was assessed via a χ^2^ test (chi-squared, where *p* < 0.10 indicates significant heterogeneity) and the I^2^ (degree of heterogeneity) statistic. Publication bias was assessed via visual inspection of the funnel plot.

## 7. Results of the Meta-Analysis

### 7.1. Study Selection

**Figure 2:**
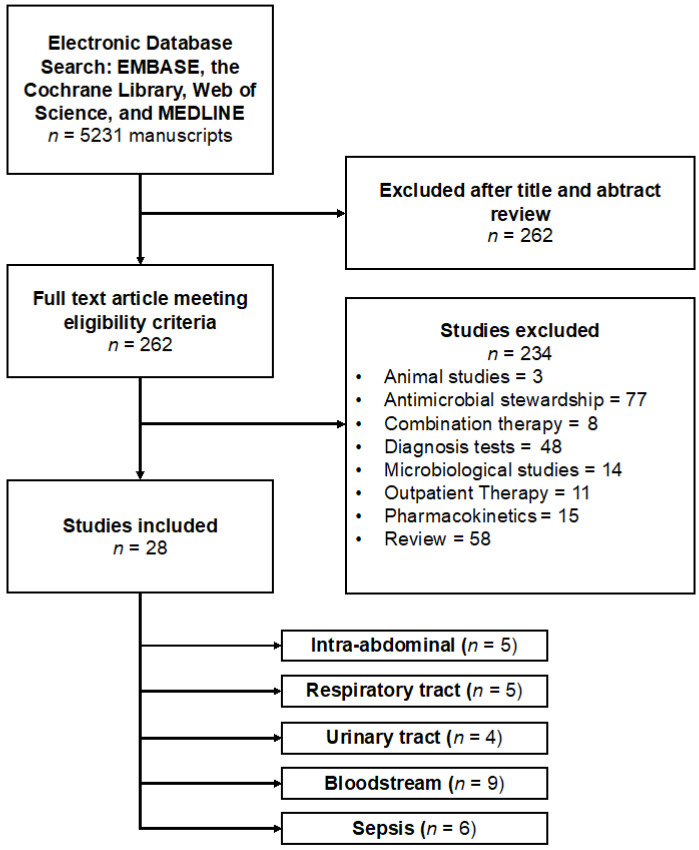
Flowchart of studies included in the meta-analysis to evaluate adequate antimicrobial therapy and mortality.

The twenty-eight included studies contained a total of 46,885 participants, with a median of 345 participants per study, a minimum of 28 participants, and a maximum of 35,529 participants [53,54,55,56,57,58,59,60,61,62,63,64,65,66,67,68,69,70,71,72,73,74,75,76,77,78]. On Cohen’s kappa scale, there was a 0.92 agreement between the two reviewers. After conducting a full-text search, we included 28 papers. Figure 2 depicts reporting items for systematic reviews and meta-analyses (PRISMA) study selection strategy.

### 7.2. Study Characteristics

**Figure 3:**
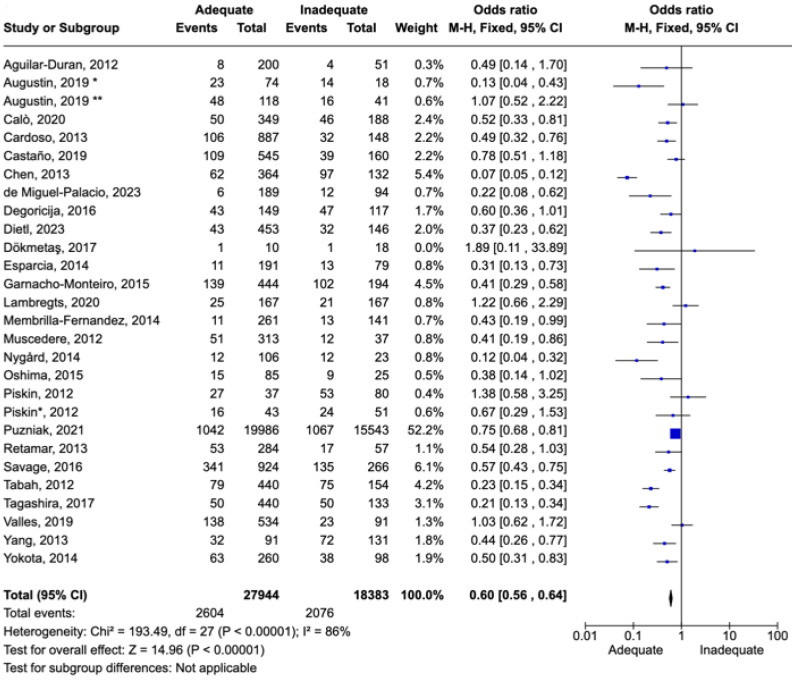
Forest plot of studies included in the meta-analysis to evaluate adequate antimicrobial therapy and mortality in different infection sites (abdominal, respiratory, urinary, bloodstream, and sepsis). * Patients admitted on intensive care unit. ** Patients admitted on ward [53,54,55,56,57,58,59,60,61,62,63,64,65,66,67,68,69,70,71,72,73,74,75,76,77,78].

The twenty-eight included studies contained a total of 46,885 participants, with a median of 345 participants per study, a minimum of 28 participants, and a maximum of 35,529 participants. Among the sites of infection, the most studied were bloodstream infections (*n* = 9), followed by respiratory tract infections (*n* = 5), intra-abdominal infections (*n* = 5), and urinary tract infections, evaluated by 3 studies. Studies that exclusively included patients with sepsis and septic shock were evaluated separately due to severity, although primary sites of sepsis were not included in studies that evaluated site-specific infection (Figure 3).

### 7.3. Results of All Infection Sites

Among the sites of infection, the most studied were bloodstream infections (*n* = 9), followed by respiratory tract infections (*n* = 5), intra-abdominal infections (*n* = 5), and urinary tract infections, evaluated by 3 studies. Studies that exclusively included patients with sepsis and septic shock were evaluated separately due to severity, although primary sites of sepsis were not included in studies that evaluated site-specific infection.

All studies were retrospective, involving adult patients with mortality as the primary outcome. Mortality endpoints varied across studies, with some using 30-day mortality (*n* = 19) and others assessing in-hospital mortality. Preference was given to the analysis of 30-day mortality when both endpoints were available to mitigate the effects of delayed mortality due to causes other than infection, although infection may have contributed to prolonged hospitalization.

Four studies were conducted exclusively in emergency rooms, seven in ICUs, three in general wards, and the remainder in a combination of these settings. While all patients were hospitalized at the time of antibiotic prescription, some infections were defined as community-acquired (eight studies), while the majority were healthcare-associated infections. The number of patients included was 46,327. There were 4680 deaths, resulting in a mortality rate of 10.1%. Adequate therapy was associated with reduced mortality, with an odds ratio (OR) of 0.60 [0.56–0.64] (Figure 3).

### 7.4. Abdominal Infections

**Figure 4:**
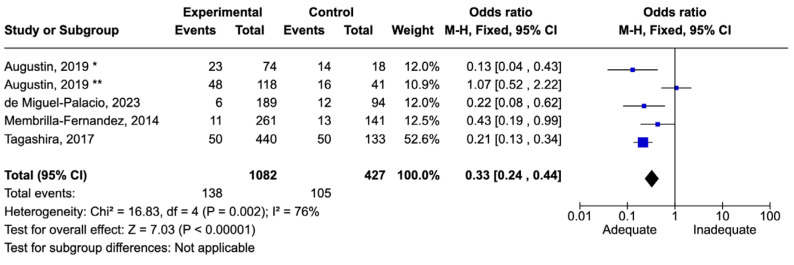
Forest plot of studies included in the meta-analysis to evaluate adequate antimicrobial therapy and mortality in intra-abdominal infections. * Patients admitted on intensive care unit. ** Patients admitted on ward [65,69,73,78].

The number of patients with abdominal infections was 1509. The nature of the infections varied from secondary peritonitis to cholecystitis, with subgroup classification by infection type and severity not feasible. There were 243 deaths, corresponding to a mortality rate of 16.1%. Adequate therapy was associated with reduced mortality, with an odds ratio (OR) of 0.33 [0.24–0.44] (Figure 4).

### 7.5. Bloodstream Infections

**Figure 5:**
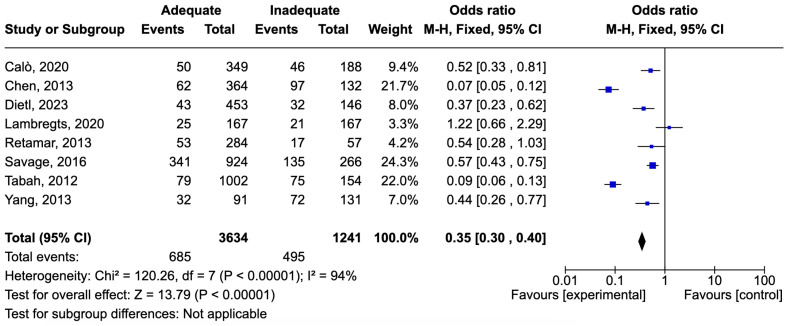
Forest plot of studies included in the meta-analysis to evaluate adequate antimicrobial therapy and mortality in bloodstream infections [56,58,60,61,68,74,75,77].

The definition of bloodstream infections (BSIs) was consistent across the studies, with most studies categorizing infections based on mandatory microorganism identification criteria as per CDC guidelines. The microbiological profile was based on non-species-specific studies and also those targeting pathogens like *Pseudomonas aeruginosa* and ESBL-producing *Enterobacterales*. The number of patients with BSIs was 4875. There were 1180 deaths, resulting in a mortality rate of 24.2%. Adequate therapy was associated with reduced mortality, with an odds ratio (OR) of 0.35 [0.30–0.40] (Figure 5).

### 7.6. Respiratory Tract Infections

**Figure 6:**
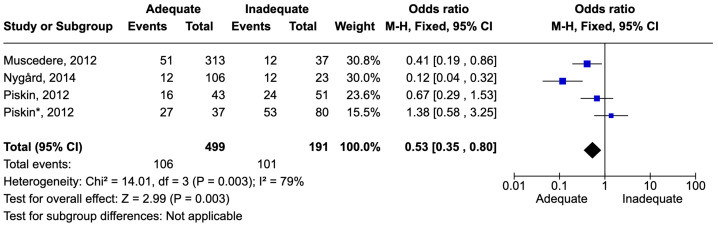
Forest plot of studies included in the meta-analysis to evaluate adequate antimicrobial therapy and mortality in respiratory tract infections. * Patients admitted on intensive care unit [54,57,62].

The definition of respiratory tract infections (RTIs) was generally consistent across the studies. However, these included community-acquired as well as hospital-acquired infections, including ventilator-associated pneumonia. The number of patients with RTIs was 690. There were 207 deaths, resulting in a mortality rate of 30.0%. Adequate therapy was associated with reduced mortality, with an odds ratio (OR) of 0.53 [0.35–0.80] (Figure 6).

### 7.7. Urinary Tract Infections

**Figure 7:**
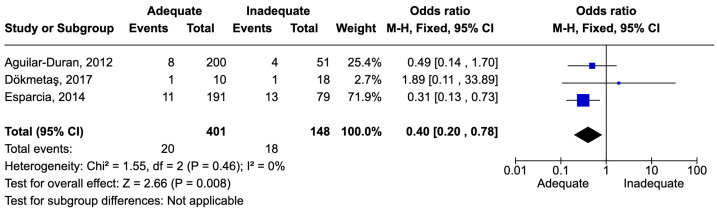
Forest plot of studies included in the meta-analysis to evaluate adequate antimicrobial therapy and mortality in urinary tract infections [55,63,70].

The definition of urinary tract infections was consistent with pyelonephritis and catheter-associated infections. The number of patients with UTIs was 549. There were 38 deaths, resulting in a mortality rate of 6.9%. Adequate therapy was associated with reduced mortality, with an odds ratio (OR) of 0.40 [0.20–0.78] (Figure 7).

### 7.8. Sepsis and Septic Shock

**Figure 8:**
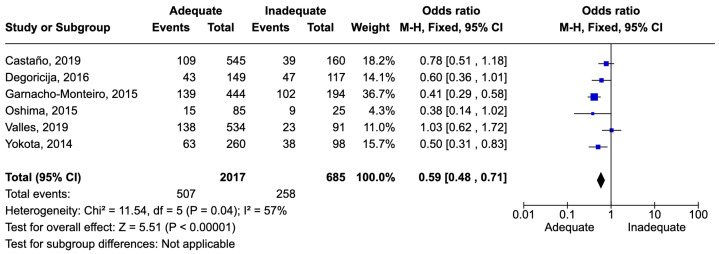
Forest plot of studies included in the meta-analysis to evaluate adequate antimicrobial therapy and mortality in sepsis [53,64,66,67,71,72].

The number of patients with sepsis or septic shock was 2702. There were 765 deaths, corresponding to a mortality rate of 28.3%. Adequate therapy was associated with reduced mortality, with an odds ratio (OR) of 0.59 [0.48–0.71] (Figure 8).

### 7.9. Results of Syntheses

All studies were retrospective, as it would be ethically impossible to propose inadequate therapy or to maintain it after pathogen identification. Among the biases encountered, the primary issue was an imbalance in cases, favoring a larger number of patients from the Puzniak et al. study [76]. The etiologies of infection varied across studies, and it was not feasible to separate them by pathogens, knowing that the severity of each infection varies depending on the pathogens involved.

## 8. Discussion

**Figure 9:**
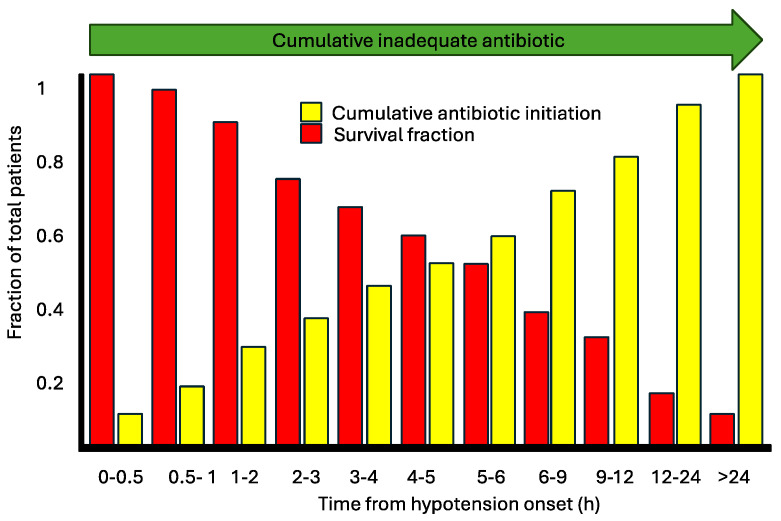
Correlation between mortality and cumulative time of inadequate antibiotic (adapted from Kumar et al., 2006) [80].

Mortality increases significantly when empirical antibiotics are not adequate. The risk of death increases by 1.7 (70%), showing the importance of the rapid identification of bacteria or correct empirical antimicrobial therapy based on concrete epidemiological data. The correct empirical therapy is the most effective approach to reduce mortality, emphasizing the importance of initiating treatment as early as possible [79]. One of the seminal works illustrating the association between timing and appropriate therapy was published by Kumar et al., in 2006, focusing on patients with infection and associated hypotension, which would now be categorized as sepsis and septic shock [80]. According to this study, each hour of delay in appropriate therapy corresponded to an increase in mortality proportionally. We consider delayed appropriate therapy in the same interpretive framework as Kumar et al.’s study (Figure 9). However, we lack studies specifically evaluating the impact of hours of delay in appropriate versus inappropriate therapy. What we can assert is that inappropriate therapy within 24 h is associated with higher mortality [81].

This systematic review clearly demonstrates that for all the infection sites included, mortality decreases with appropriate therapy, even for infections traditionally considered less severe such as urinary tract infections. Interestingly, while mortality rates in respiratory tract infections were noted, we did not identify a clear reason for the higher mortality in RTIs compared to sepsis, where one might expect higher mortality [82]. In one study of RTIs, patients were admitted to the ICU, whereas in another study, they were already in the ICU. In a study including community-acquired pneumonia patients, the severity criteria were met but a definition of sepsis was not provided, thus excluding them from the sepsis group. Conversely, in studies involving sepsis patients, potentially lower mortality rates were attributed to the faster initiation of therapy, as sepsis protocols demanding broad-spectrum therapy within the first hour (bundles) are stringent in this regard [82]. Additionally, the time from symptom onset to therapy initiation is typically longer for community-acquired pneumonia patients who present from home.

In bloodstream infection studies, there was a predominance of specific pathogen etiology investigations. Consequently, we could not determine superior severity or mortality outcomes among the studies. As previously described, mortality associated with inadequate therapy in hospital-acquired bloodstream infections is higher in pathogens like *Acinetobacter baumannii*, extended-spectrum beta-lactamase-producing *Enterobacteriaceae*, and carbapenemase-producing KPC types [81,83,84].

Another crucial variable to consider is the antibiotic class used in treating these patients, as pharmacokinetic and pharmacodynamic factors can influence clinical response, despite conflicting views in the literature. Adequate therapy should not solely consider antibiotic spectrum coverage against the pathogen; factors such as tissue penetration, dosage, and adverse events are equally important [19,85].

Of particular interest is the potential 50% reduction in mortality risk attributed to appropriate therapy. However, an analysis of hospitalization duration and costs could not be included as a sub-analysis in this meta-analysis but could provide additional insights into patient quality of life, acquisition of multidrug-resistant bacteria, and costs associated with prolonged hospital stays, as demonstrated in various studies [21].

Presently, healthcare systems’ sustainability is increasingly being scrutinized, especially in developing countries’ hospitals. By conserving financial resources, investments can be made in enhanced diagnostic capabilities to expedite pathogen identification, thereby increasing diagnostic accuracy and reducing mortality. In this context, the application of molecular panels directly from blood or specific infection sites enables rapid pathogen identification, reinforcing the importance of timely, appropriate therapy within 24 h.

However, the mere fact of rapid identification is not sufficient. Several studies demonstrate that without an effective antimicrobial stewardship team, the implementation of diagnostic technologies may not achieve the desired effect [86,87]. In addition to the “aggressive” activity of antimicrobial stewardship programs for adjusting, de-escalating, or even discontinuing unnecessary antibiotics, not all hospitals have the capability to maintain a full team of dedicated professionals for antimicrobial care [25]. In this regard, the implementation of tele-stewardship is appealing, as it allows for cost reductions in staffing and integrates real-time technologies with diagnostic resources.

This is a meta-analysis, and classical limitations must be considered. Many limitations were described during the discussion, but retrospective studies have a significant impact. However, it would not be possible to conduct prospective studies on antimicrobial error. The doses of antibiotics were not considered, nor were the specific antibiotics used and the duration of treatment. The severity of the patients was not assessable due to the differences between the studies, nor was the matching of groups by age and comorbidities. Although one study had a larger number of patients than the others, the trend in reduced mortality was consistent across the other publications. Thus, we conclude that there was significant heterogeneity. Publication bias is an important factor, as it is uncommon for studies to show increased mortality with appropriate therapy, and few have shown no difference. Among the comparability biases, the mortality outcome varied between 30-day mortality and total hospital mortality. The clinical response variable would be the best way to analyze the antimicrobial response, but this information is rarely used in publications due to its variability in clinical interpretation. A variable not found in these studies was the length of hospital stay in patients with inadequate versus adequate therapy. With this variable, we could also include a cost–benefit analysis of rapid diagnosis.

## 9. Conclusions

Early appropriate therapy within the first 24 h is associated with reduced mortality in respiratory, urinary, abdominal, and bloodstream infections, and cases of sepsis. In this context, we highlight that healthcare technologies aimed at minimizing therapeutic inadequacies contribute to mortality reduction and, by extension, facilitate shorter hospital stays and decreased healthcare costs. These savings enable investments in antimicrobial stewardship programs and rapid diagnostic laboratory initiatives.

## Data Availability

Data are available under request.

## Supplementary Materials

The following supporting information can be downloaded at: https://www.mdpi.com/article/10.3390/idr16040054/s1, Table S1: PRISMA 2020 Checklist.
